# Development and evaluation of an efficient and real-time monitoring system for the vector mosquitoes, *Aedes albopictus* and *Culex quinquefasciatus*

**DOI:** 10.1371/journal.pntd.0010701

**Published:** 2022-09-08

**Authors:** Zetian Lai, Jing Wu, Xiaolin Xiao, Lihua Xie, Tong Liu, Jingni Zhou, Ye Xu, Yiquan Cai, Feng Lin, Bin Li, Lu Gan, Anthony A. James, Xiao-Guang Chen

**Affiliations:** 1 Department of Pathogen Biology, Institute of Tropical Medicine, School of Public Health, Southern Medical University, Guangzhou, China; 2 Guangzhou NewVision Electronic and Technology Co. Ltd, Guangzhou, China; 3 Guangdong Huilimin Pest Control Engineering Co. Ltd, Guangzhou, China; 4 Departments of Microbiology & Molecular Genetics and Molecular Biology & Biochemistry, University of California, Irvine, California, United States of America; University of Florida, UNITED STATES

## Abstract

**Background:**

The surveillance of vector mosquitoes is essential for prevention and control of mosquito-borne diseases. In this study, we developed an internet-based vector mosquito monitor, MS-300, and evaluated its efficiency for the capture of the important vector mosquitoes, *Aedes albopictus* and *Culex quinquefasciatus*, in laboratory and field trials.

**Methodology/Principal findings:**

The linear sizes of adult *Ae*. *albopictus* and *Cx*. *quinquefasciatus* were measured and an infrared window was designed based on these data. A device to specifically attract these two species and automatically transmit the number of captured mosquitoes to the internet was developed. The efficiency of the device in capturing the two species was tested in laboratory, semi-field and open field trials. The efficiency results for MS-300 for catching and identifying *Ae*. *albopictus* in laboratory mosquito-net cages were 98.5% and 99.3%, and 95.8% and 98.6%, respectively, for *Cx*. *quinquefasciatus*. In a wire-gauze screened house in semi-field trials, the efficiencies of MS-300 baited with a lure in catching *Ae*. *albopictus* and *Cx*. *quinquefasciatus* were 54.2% and 51.3%, respectively, which were significantly higher than 4% and 4.2% without the lure. The real-time monitoring data revealed two daily activity peaks for *Ae*. *albopictus* (8:00–10:00 and 17:00–19:00), and one peak for *Cx*. *quinquefasciatus* (20:00–24:00). During a 98-day surveillance trial in the field, totals of 1,118 *Ae*. *albopictus* and 2,302 *Cx*. *quinquefasciatus* were captured by MS-300. There is a close correlation between the number of captured mosquitoes and the temperature in the field, and a positive correlation in the species composition of the captured samples among the mosquitoes using MS-300, BioGents Sentinel traps and human landing catches.

**Conclusions/Significance:**

The data support the conclusion that MS-300 can specifically and efficiently capture *Ae*. *albopictus* and *Cx*. *quinquefasciatus*, and monitor their density automatically in real-time. Therefore, MS-300 has potential for use as a surveillance tool for prevention and control of vector mosquitoes.

## Author summary

Surveillance of vector mosquitoes is essential for prevention and control of mosquito-borne diseases. However, traditional methods for monitoring mosquitoes may not meet ethical requirements and are labor-intensive. We developed an internet-based vector mosquito monitor, MS-300, that can sensitively detect mosquito entry and automatically transmit the data to cloud-based server services and remote terminals. We evaluated its efficiency for the capture of the important vector mosquitoes, *Aedes albopictus* and *Culex quinquefasciatus*, in laboratory, semi-field and field trials. Our results demonstrate that the MS-300 device could monitor *Ae*. *albopictus* and *Cx*. *quinquefasciatus* with results comparable with human landing catches and BioGents Sentinel traps. The MS-300 monitor can be a dependable tool for assessing *Ae*. *albopictus* and *Cx*. *quinquefasciatus* population densities and circadian activity patterns in both laboratory and field surveys and can be used to guide prevention and control efforts of mosquito-borne diseases when applied to daily and seasonal population fluctuations.

## Introduction

Mosquito-borne diseases such as malaria, filariasis and dengue fever have placed a huge burden on human populations worldwide [1–3]. *Aedes albopictus* is a major vector of the viruses that cause dengue fever and Zika virus infections [4]. *Culex quinquefasciatus* is the primary vector for the viral and filarial pathogens that cause Saint Louis encephalitis, West Nile fever, Rift Valley fever and lymphatic filariasis [5–8]. *Aedes albopictus* and *Cx*. *quinquefasciatus* are the predominant mosquito species in urban China [9, 10]. At present, integrated mosquito management is the main strategy for prevention and control of vector mosquitoes and the pathogens they transmit [11].

Monitoring the density of vector mosquitoes is important for predicting possible epidemics of mosquito-borne infectious diseases and evaluating vector control. At present, the methods for monitoring adult mosquito densities are light traps, human-baited double-net traps, BioGents Sentinel (BGS) traps and human landing catches (HLCs) [12–14]. However, light traps for monitoring *Ae*. *albopictus* have limited efficacy because they are principally active diurnally [14, 15]. Traditional HLC methods may not meet ethical requirements and have potential health risks for collectors during an epidemic. Human-baited, double-net trap are an alternative to HLCs for collecting outdoor vector mosquitoes but they are labor-intensive and time-consuming [13].

BGS traps have been used widely and are reported to be effective when applied to monitoring and estimating vector mosquito densities [16–19]. However, these traps still require manual support to count and identify the captured mosquitoes and may not reflect mosquito densities in a timely manner. Recently, a new device from BioGents, named the BG-Counter, was developed to count automatically captured mosquitoes and transfer the data remotely to the surveillance team. However, the reported sensitivity and specificity of the BG-Counter was shown to vary greatly in a study comprising five North Carolina counties with mean daily accuracies ranging from 9.4% to 80.1% [20]. Therefore, although the BG-Counter may be a useful alternative in some circumstances, the accuracy will need to be improved before is should be widely adopted.

We designed a next-generation trap, MS-300, for *Ae*. *albopictus* and *Cx*. *quinquefasciatus* that contains an infrared window and automatic counting device to sensitively detect mosquito entry and automatically transmit the data to cloud-based server and remote terminals. An environmental sensor in MS-300 can monitor the relationships among mosquito densities and daily and seasonal factors. The efficiencies of the device for capturing *Ae*. *albopictus* and *Cx*. *quinquefasciatus* were evaluated in a mosquito net cage, wire-gauze screened house and in the field, and analyzed in comparison with data from BGS trap and HLCs.

## Material and methods

### Ethics statement

No specific permits were required for the described field studies. These studies did not involve endangered or protected species. Collectors provided written consent to participate in the human landing catches.

### Mosquitoes

Established laboratory colonies of *Ae*. *albopictus* and *Cx*. *quinquefasciatus* collected from Foshan (in 1981) and Guangzhou (in 1993), Guangdong Province, China, were provided by the Guangdong Provincial Center for Disease Control and Prevention. Larvae (120–200 larvae/L water) were reared in stainless-steel trays containing dechlorinated water and were fed yeast and turtle food. All mosquito stages were reared in an insectary maintained at 27±1°C, 70%–80% relative humidity and a 16-h light/8-h dark photoperiod, and adults offered a 10% sucrose solution *ad libitum*.

### Mosquito size measurements

After anesthesia with ice, the linear lengths of each of 50 5–7 day-old adult male and female *Ae*. *albopictus* and *Cx*. *quinquefasciatus* were measured using stereomicroscopy from a lateral view (S1 Fig). The average lengths from the top of the head to the tip of the genitalia were recorded as a size reference for designing the infrared grates to identify the passing objects.

### Setting the infrared detection window

The average lengths of the target mosquitoes were recorded as a reference for designing the X- and Y-dimensions of the infrared grates. When the objects passed through the infrared detection window, the projection lines in X and Y dimensions would be blocked. If the size of the passing object matches with the reference, the data would be recognized and recorded as one of the target mosquitoes. Because environmental illumination may affect the emission and reception of infrared, the standard for the infrared grates needs to be adjusted accordingly to the light conditions of the study sites. The passage time of the object through the detection window is varied based on the size and flying speed of target mosquito and the speed of the fan in the MS-300 monitor, so that the infrared scanning intervals were set accordingly to such specific data. When an object was trapped by the MS-300, the passing data are recorded and analyzed to determine whether or not the object is a mosquito.

### Assembling the MS-300 monitor

The MS-300 device comprises components that include a mosquito detection platform, data processing hardware, collection bag, fan and lure. The mosquito lure, Mix-5, is a proprietary reagent and efficiently attracts *Ae*. *albopictus* and *Cx*. *quinquefasciatus* [21]. The environmental sensor records the temperature and illumination of the study site every 30 minutes.

### MS-300 monitoring system

The MS-300 monitoring system is supported by a web-based database for storage of mosquito counts, geospatial and environmental data (temperature and illumination). The data are transferred to an internet web page that can be accessed via PC, smartphone or tablet. Using a 4G wireless communication network and the web application, investigators can manage the MS-300 monitor and collect data on mosquito population dynamics, daily activity patterns and environmental factors.

### Laboratory tests

The MS-300 was placed in a cage constructed of mosquito netting (3 × 2 ×2m). A total of 100 female *Ae*. *albopictus* or *Cx*. *quinquefasciatus* 5–7 days post-emergence were released into the cage. The mosquitoes captured in the mosquito bag server were counted after 24 hours and compared with the data sent to the cloud-based server. The experiment was repeated six times. Efficiency and accuracy were calculated as follows:

Eq 1: Efficiency = number of mosquitoes in bag/number of mosquitoes released.

Eq 2: Accuracy = number of mosquitoes in bag/cloud-based server data.

When the number of mosquitoes in the bag was more than that reported in the cloud-based server data, an absolute value in the following formula was used:

Eq 3: Correction formula of accuracy = 1-∣[1-(number of mosquitoes in bag/cloud-based server data)]∣*100.

### Semi-field tests

Two MS-300 monitors, one with and one without lure, were placed parallel in the wire-gauze screened house (4 × 3 × 2.1m). One hundred female *Ae*. *albopictus* or *Cx*. *quinquefasciatus* were released into the house. The mosquitoes trapped in the MS-300 bag as well as the numbers sent to the cloud-based server data were counted 24 hours later. The experiment was repeated six times. Efficiency and accuracy were calculated using the formulae described above.

### Field tests

One MS-300 monitor and one BGS trap were placed (5 meters apart) in a garden (outdoor) or a residence (indoor). The Mix-5 lures were used in the absence of CO_2_. The insects trapped into the collection bag as well as the cloud-based server data were counted every 24 hours. The experiment was conducted for 98 days (From May 1, 2021 to August 14, 2021). HLCs were conducted at 17:30–18:30 every seven days based on the standard manual [22]. The formulas were calculated as follows:

Eq 1: Accuracy = number of mosquitoes in bag/cloud-based server data.

Eq 2: Specificity = number of mosquitoes in bag/number of insects in bag.

Eq 3: HLCs = number of mosquitoes collected/(person*hour).

### Statistical analyses

All statistical analyses were performed using SPSS version 20.0 (IBM, Chicago, IL, USA). The efficiency, accuracy and specificity of the devices were compared using independent samples t-tests or nonparametric tests. The number of mosquitoes in the MS-300 monitor and BGS traps were compared using a nonparametric test. Pearson correlation coefficient, r, was used to determine the strength and direction of a linear relationship, between two variables [23, 24]. The correlation between the MS-300 monitor and temperature were determined by Pearson’s correlation analysis test. Pearson’s correlation analysis test also was used to determine if there was an association among MS-300 monitors, BGS traps and HLCs. *P*-values > 0.05 were considered not significant.

## Results

### Sizes of adult male and female *Ae*. *albopictus* and *Cx*. *quinquefasciatus*

Fifty male and female mosquitoes each were measured using stereomicroscopy (S1 Fig). The average lengths of *Ae*. *albopictus* were 4.75 ± 0.27mm for the females and 3.99 ± 0.11mm for the males, and those of *Cx*. *quinquefasciatus* were 4.83 ± 0.30mm for the females and 4.62 ± 0.30mm for the males (S1 Table).

### Development of the specific and real-time monitor for *Ae*. *albopictus* and *Cx*. *quinquefasciatus*

**Fig 1:**
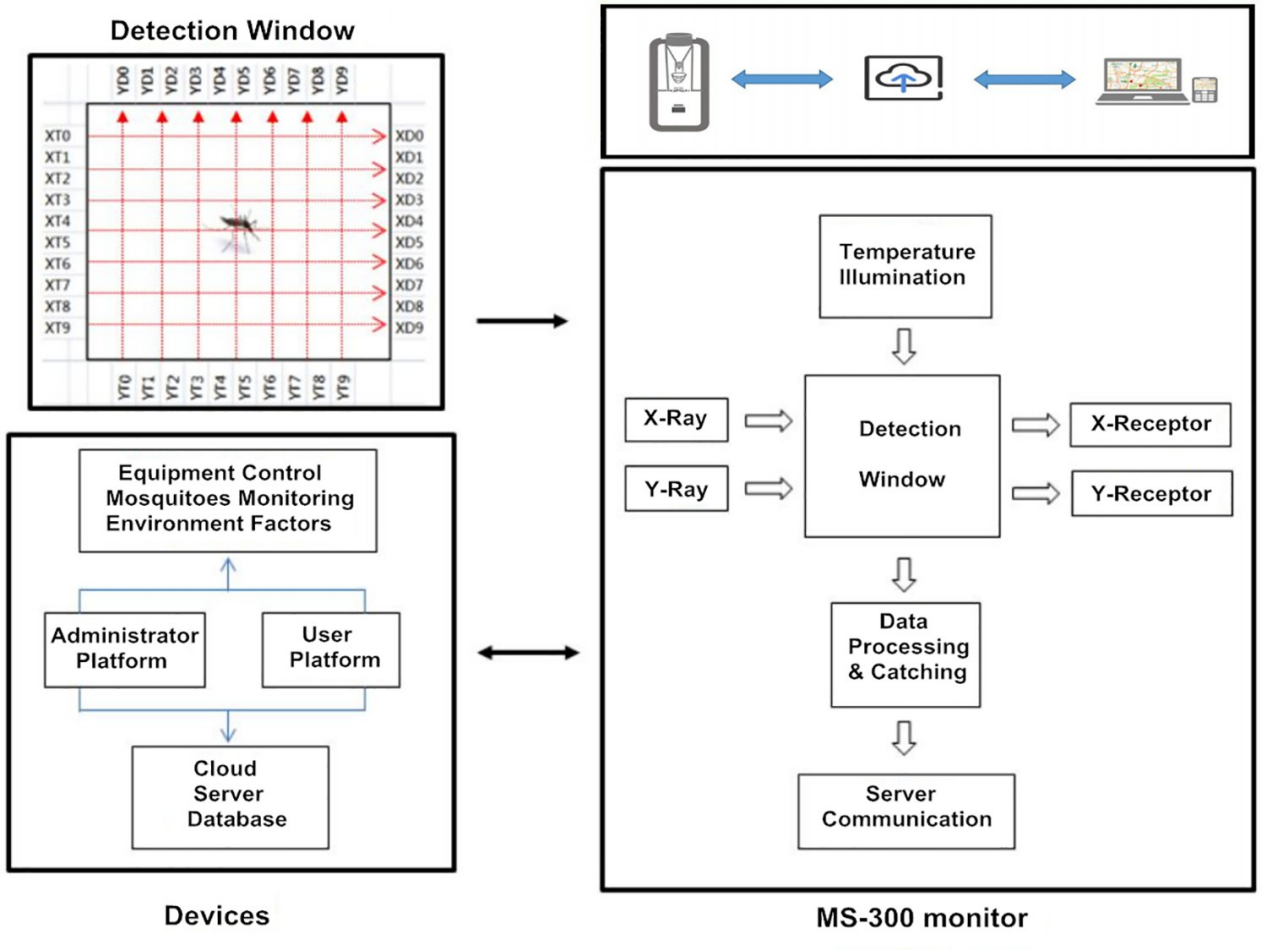
Schematic diagram of the detection and data transmission modules of MS-300. The MS-300 monitor contains an infrared detection window and automatic counting device. An environmental sensor monitors and records the environmental factors such as temperature and illumination. Data processing hardware and software in the monitor perform an analysis and real-time data are transmitted automatically to cloud-based server services and remote terminals.

The MS-300 monitor includes the infrared probes and automatic counting devices (Fig 1). The X- and Y-dimension infrared grates were set based on the sizes of *Ae*. *albopictus* and *Cx*. *quinquefasciatus* (S1 Table).

**Fig 2:**
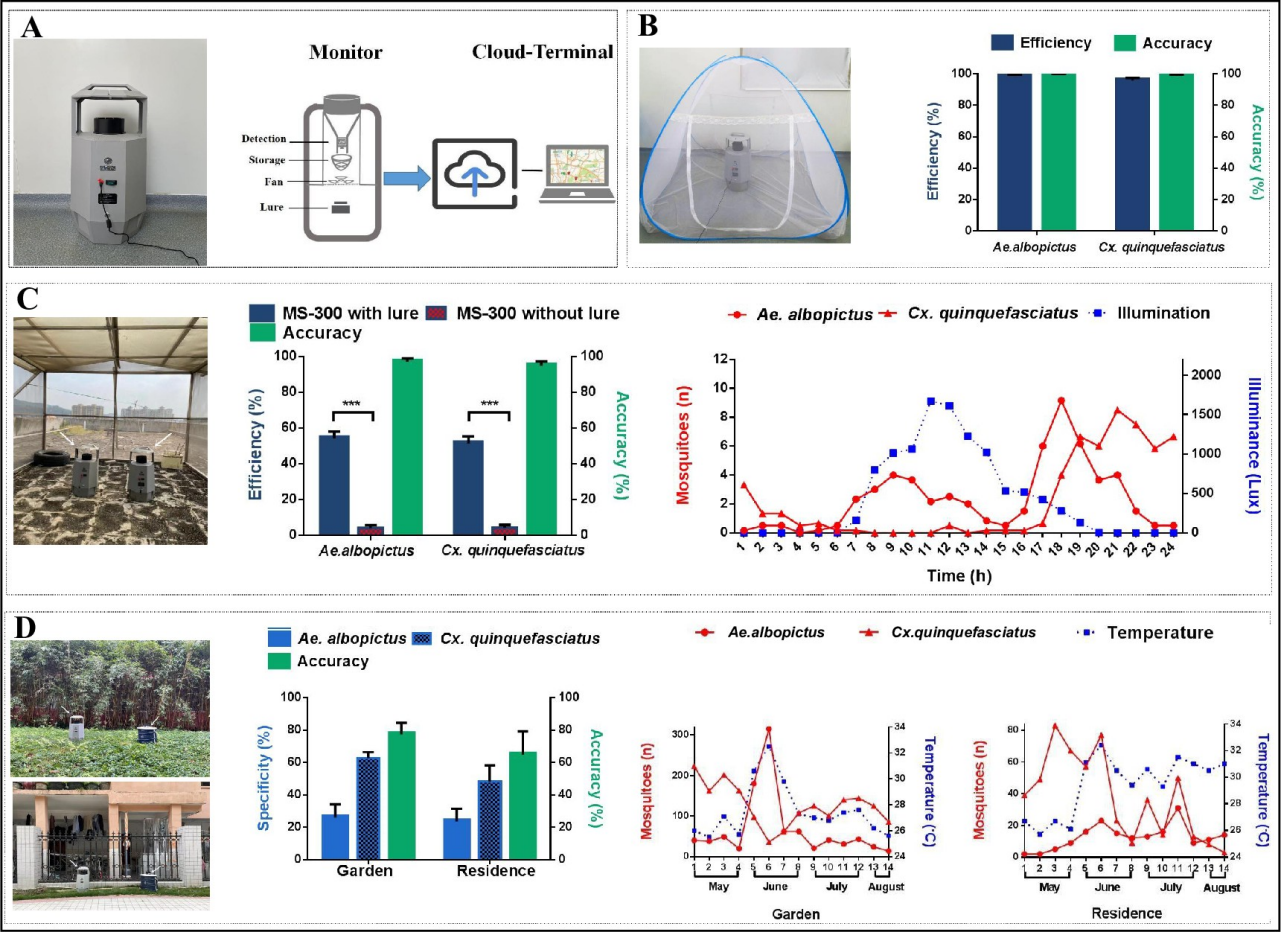
Test of MS-300 monitor in different settings. **(**A) Left: Photograph of the MS-300 monitor; Right: Schematic diagram of MS-300 monitor. (B) Test of MS-300 monitor in laboratory. Left: actual laboratory scene; Right: efficiency and accuracy verification of the MS-300 monitor in capturing mosquitoes; the error bars represent 95% confidence intervals (CIs). **(**C) Test of MS-300 monitor in semi-field. Left: actual semi-field scene; Center: efficiency and accuracy verification of the MS-300 monitor in capturing mosquitoes; Right: hourly mosquito capture numbers over 24 hours; the error bars represent 95% CIs; ***, P < 0.001. **(**D) Test of the MS-300 monitor in field. Left: actual field scene; Center: specificity of the MS-300 monitor in catching *Ae*. *albopictus* and *Cx*. *quinquefasciatus* in two sites; Right: weekly variations in the densities of *Ae*. *albopictus* and *Cx*. *quinquefasciatus* and local temperatures in study sites; the error bars represent 95% CIs.

The MS-300 monitor combines components that include a mosquito detection platform, data processing hardware and software, a collection bag, fan and the Mix-5 lure (Fig 2A). The gelatinous lure is a patented attractant and is efficient for attracting *Ae*. *albopictus* and *Cx*. *quinquefasciatus*, therefore the MS-300 monitor does not require the use of large CO_2_ canisters for attraction. When the device is running, airflow will volatilize the Mix-5. The mosquitoes are attracted by Mix-5 and suctioned by a fan into the monitor. The objects passing through the detection device are differentiated from other insects or dust particles as mosquitoes and recorded. The mosquito data are transferred automatically to a cloud-based server at 30-min intervals and accessed via a PC, smartphone or tablet in real-time. Furthermore, local environmental factors such as temperature and illumination can be sampled and analyzed with the mosquito density in real-time.

### Test of MS-300 monitor in the laboratory

One hundred female *Ae*. *albopictus* or *Cx*. *quinquefasciatus* were released into the mosquito net cage and the numbers of mosquitoes in the MS-300 collection bag and reported to the cloud-based server were counted 24h later (Fig 2B, left). The efficiency and accuracy of the MS-300 monitor in catching and identifying *Ae*. *albopictus* were 98.5% and 99.3%, respectively, and 95.8% and 98.6% in catching *Cx*. *quinquefasciatus* (Fig 2B, right). An average of 98.5±1.5 *Ae*. *albopictus* were captured by MS-300 and 99.2±1.2 were recorded in the cloud-based server, while 95.8±2.6 and 97.2±2.4, respectively, were recorded for *Cx*. *quinquefasciatus* (S2 Table). No significant differences are detected in efficiency and identification between the *Ae*. *albopictus* and *Cx*. *quinquefasciatus* captured by the MS-300 monitor and this supports the conclusion that the monitor can accurately identify and record these two species.

### Test of the MS-300 monitor in semi-field

Two MS-300 monitors, one with lure (treatment) and one without (control), were placed in parallel in a wire-gauze screen house (4m × 2m × 2.1m) (Fig 2C, left). One hundred female *Ae*. *albopictus* or *Cx*. *quinquefasciatus* were released into the enclosure and the numbers of mosquitoes in the MS-300 collection bag and in cloud-based server were counted 24h later. The efficiencies of the treatment in catching *Ae*. *albopictus* and *Cx*. *quinquefasciatus* were 54.2% and 51.3%, respectively, which are significantly higher than 4% and 4.2% of the control (t = 9.51, *p*<0.001; t = 12.314, *p*<0.001) (Fig 2C, center), indicating that Mix-5 was effective in attracting *Ae*. *albopictus* and *Cx*. *quinquefasciatus*. No significant differences in the accuracy of the MS-300 monitor were found with *Ae*. *albopictus* and *Cx*. *quinquefasciatus* (96.3% and 95.1%, respectively, S3 Table), and this supports the conclusion that the monitor can accurately identify and record *Ae*. *albopictus* and *Cx*. *quinquefasciatus*. An average of 41.8±12.9 *Ae*. *albopictus* and 44.5±10 *Cx*. *quinquefasciatus* were uncaptured, which included dead mosquitoes on the floor of the structure (S3 Table).

The numbers of *Ae*. *albopictus* and *Cx*. *quinquefasciatus* captured in the MS-300 monitor and recorded in the cloud-based server and illumination at one-hour intervals for a total of 24 hours also were recorded (Fig 2C, right and S3 Table). The time span of illumination was 7:00–19:00 with peak illuminance (1673 lux) at 11:00. The illuminance gradually decreased and dropped to 0 at 20:00. The first captured peak in *Ae*. *albopictus* showed a slow increase and then remain stable (8:00–10:00), while the second peak showed a rapid increase with an obvious pattern (17:00–19:00). An average of 4±0.6 (maximum) *Ae*. *albopictus* were captured at 09:00 (1011 lux) in the first active period, while 9.2±2.9 (maximum) were recorded at 18:00 (278 lux) in the second active period. Only one active period (19:00–24:00) was recorded in *Cx*. *quinquefasciatus* with an average of 8.5±1.6 (maximum) at 21:00 (0 lux). These results support the conclusion that MS-300 monitor can accurately monitor the daily activity *Ae*. *albopictus* and *Cx*. *quinquefasciatus* (Fig 2C, right).

### Test of MS-300 monitor in the field

Field tests of MS-300 monitor were conducted from May 1 to August 14, 2021, in a garden and a residence using BGS traps as controls (Fig 2D, left). A total of 940 and 3605 *Ae*. *albopictus* (z = -2.115, p<0.05) and 1774 and 6200 *Cx*. *quinquefasciatus* (z = -3.768, p<0.001) were trapped in the garden by MS-300 and BGS, respectively (Table 1). One-hundred, seventy-eight (178) and 2049 *Ae*. *albopictus* (z = -4.319, p<0.001) and 528 and 4553 *Cx*. *quinquefasciatus* (z = -4.069, p<0.001) were recovered in the residence by MS-300 and BGS, respectively (Table 1). These data show that the efficiency of MS-300 is significantly lower than that of BGS. However, the average specificities of MS-300 and BGS were 88.9% and 86.9%, respectively, in the garden and 71.9% and 76.8% in the residence with no significant difference between them (Table 1). In the garden, a total of 467 nontarget arthropods, accounting for 11.1% of the total insects caught, collected by MS-300 during that period, included Diptera (*Drosophila melanogaster* and *Chironomus kiiensis*), Hymenoptera and Coleoptera. In the residence, we collected 269 nontarget arthropods, accounting for 28.1% of the total insects caught by MS-300, and these were mostly species in the orders Diptera (*Drosophila melanogaster*, *Chironomus kiiensis* and *Chrysomya megacephala*), Hymenoptera, Araneae and Coleoptera (Table 1). Furthermore, no significant difference was found in the specificity of *Ae*. *albopictus* (26.1% and 23.7%) and *Cx*. *quinquefasciatus* (62.7% and 48.2%) of MS-300 monitor in the garden and residence (Fig 2D, center), and no significant differences were found in the mean accuracy (79.4% and 64.9%) of the MS-300 monitor in two field sites (Table 1).

**Table 1:** Test of three monitoring methods on *Ae*. *albopictus* and *Cx*. *quinquefasciatus* in the field.

Location	Method	Monitoring items	May.				Jun.				Jul.				Aug.		Total*
			1st-7th	8th-14th	15th-21th	22th-28th	1st-7th	8th-14th	15th-21th	22th-28th	1st-7th	8th-14th	15th-21th	22th-28th	1st-7th	8th-14th	
Garden	MS-300	No. Ae. albo	40	38	49	20	180	315	62	62	21	41	31	43	24	14	940
		No. Cx. quin quinquefasciatus	223	162	202	163	98	36	61	108	125	101	140	144	125	86	1774
		No. mosquitoes	263	200	251	183	278	351	123	170	146	142	171	187	149	100	2714
		No. other insects	13	10	98	65	141	59	2	17	15	0	33	10	4	0	467
		No. insects	276	210	349	248	419	410	125	187	161	142	204	197	153	100	3181
		Cloud-based server data	288	232	319	271	457	432	154	216	196	172	231	219	173	119	3479
		Accuracy(%)†	91.3	86.2	78.7	67.5	60.8	81.3	79.9	78.7	74.5	82.6	74	85.4	86.1	84	79.4*
		Specificity(%)¶	95.3	95.2	71.9	73.8	66.3	85.6	98.4	90.9	90.7	100	83.8	94.9	97.4	100	88.9*
	Human landing catches	No. Ae. albo	18	8	12	2	44	96	42	32	46	32	0	24	0	24	380
		No. Cx. quin quinquefasciatus	0	0	0	8	2	0	0	0	0	0	24	2	16	2	54
		HLCs §	8	6	10	2	40	96	38	32	46	32	0	22	0	6	24*
	BGS trap	No. Ae. albo	38	33	49	57	1058	1650	71	191	62	106	60	124	80	26	3605
		No. Cx. quin quinquefasciatus	921	783	668	569	299	142	292	632	312	390	252	484	369	87	6200
		No. mosquitoes	959	816	717	626	1357	1792	363	823	369	496	312	608	449	113	9800
		No. other insects	88	100	161	117	209	83	70	91	105	41	87	77	84	9	1322
		No. insects	1047	916	878	743	1566	1875	433	914	474	537	399	685	533	122	11122
		Specificity(%)¶	91.6	89.1	81.7	84.3	86.7	95.6	83.8	90	77.8	92.4	78.2	88.8	84.2	92.6	86.9*
Residence	MS-300	No. Ae. albo	2	2	5	9	16	23	15	12	13	16	31	9	11	14	178
		No. Cx. quin quinquefasciatus	39	49	83	67	57	77	23	9	36	14	50	13	8	3	528
		No. mosquitoes	41	51	88	76	73	100	38	21	49	30	81	22	19	17	706
		No. other insects	17	21	30	19	27	43	14	15	17	13	32	8	7	6	269
		No. insects	58	72	118	95	100	143	52	36	66	43	113	30	26	23	975
		Cloud-based server data	68	81	119	103	116	159	58	43	71	48	122	33	27	27	1075
		Accuracy(%)†	60.3	63	73.9	73.8	62.9	62.9	65.5	48.8	69	62.5	66.4	66.7	70.4	63	64.9*
		Specificity(%)¶	70.7	70.8	74.6	80	73	69.9	73.1	58.3	74.2	69.8	71.7	73.3	73.1	73.9	71.9*
	Human landing catches	No. Ae. albo	2	4	6	6	18	24	14	10	14	14	40	12	16	2	182
		No. Cx. quin quinquefasciatus	0	0	0	0	0	0	0	0	0	4	4	0	0	2	10
		HLCs §	2	4	6	4	18	24	12	10	14	14	34	12	12	2	12*
	BGS trap	No. Ae. albo	17	12	32	99	153	210	88	133	135	289	413	147	117	204	2049
		No. Cx. quin quinquefasciatus	417	396	502	516	381	519	300	123	251	352	460	176	109	51	4553
		No. mosquitoes	434	408	534	615	534	729	388	256	386	641	873	323	226	255	6602
		No. other insects	171	149	92	78	124	132	73	76	114	208	644	158	81	74	2174
		No. insects	605	557	626	693	658	861	461	332	500	849	1517	481	307	329	8776
		Specificity(%)¶	71.7	73.2	85.3	88.7	81.2	84.7	84.2	77.1	77.2	75.5	57.5	67.2	73.6	77.5	76.8*

* = Mean value

†Accuracy = number of mosquitoes in bag/cloud-based server data

¶Specificity = number of mosquitoes in bag/number of insects in bag

§HLCs = number of mosquitoes collected/(person*hour)

The number of *Ae*. *albopictus* captured in the garden by the MS-300 monitor showed a positive correlation with the local temperature (r = 0.891, *p*<0.01), but that was negatively-correlated for *Cx*. *quinquefasciatus* (r = -0.655, *p*<0.05) (Fig 2D, right-Garden). The number of *Ae*. *albopictus* captured in the residence by the MS-300 monitor also showed a positive correlation with the local temperature (r = 0.769, *p*<0.01), but again was negatively-correlated for *Cx*. *quinqu*efasciatus (r = -0.297, *p*>0.05), although insignificant for the latter (Fig 2D, right-Residence). While the density of mosquitoes is correlated with many factors such as temperature, humidity and rainfall, these results show that the MS-300 device could monitor the density of *Ae*. *albopictus* and *Cx*. *quinquefasciatus* in the field and correlate mosquito abundance with temperature.

**Fig 3:**
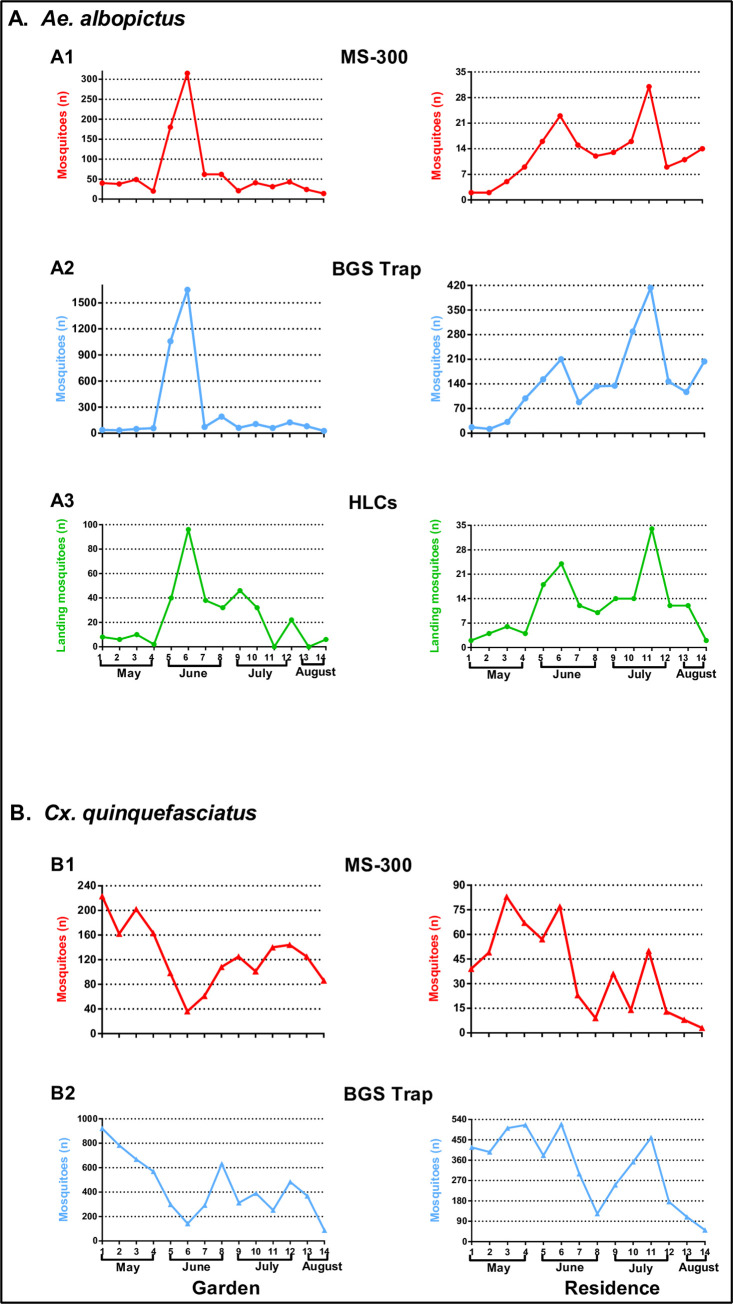
Comparisons among MS-300, BGS traps and human landing catches on the capture of *Ae*. *albopictus* and *Cx*. *quinquefasciatus*. (A) *Ae*. *albopictus*. A1: The number of *Ae*. *albopictus* captured by the MS-300 monitor at two field sites; A2: The number of *Ae*. *albopictus* captured by BGS traps at two field sites; A3: The number of *Ae*. *albopictus* captured by human landing catches at two field sites. (B) *Cx*. *quinquefasciatus*. B1: The number of *Cx*. *quinquefasciatus* captured by the MS-300 monitor at two field sites; B2: The number of *Cx*. *quinquefasciatus* captured by BGS trap at two field sites.

In addition to comparing the efficiency of the MS-300 monitor and BGS traps, HLCs were conducted every week in two study sites (Fig 3). The number of *Ae*. *albopictus* captured by HLCs in the garden were correlated significantly between the MS-300 monitor (r = 0.836, p<0.01) and BGS trap (r = 0.808, *p*<0.01). The number of *Ae*. *albopictus* trapped in the MS-300 monitor was correlated strongly to that in BGS trap (r = 0.985, *p*<0.01) (Fig 3A). For *Cx*. *quinquefasciatus*, the number captured by the MS-300 monitor also was correlated to that of the BGS traps (r = 0.814, *p*<0.01) (Fig 3B). The number of *Ae*. *albopictus* captured by HLCs in the residence was correlated significantly with the MS-300 monitor (r = 0.894, *p*<0.01) and BGS trap (r = 0.777, *p*<0.01). Furthermore, the number of *Ae*. *albopictus* in the MS-300 monitor was correlated strongly to that in the BGS trap (r = 0.899, *p*<0.01) (Fig 3A). The number of *Cx*. *quinquefasciatus* captured by the MS-300 monitor correlates with that in the BGS trap (r = 0.898, *p*<0.01) (Fig 3B). The statistical analysis showed a significant positive relationship among the MS-300 monitor, BGS trap and HLCs, which supports the conclusion that the MS-300 device could monitor *Ae*. *albopictus* and *Cx*. *quinquefasciatus* as well as HLCs and BGS trap.

## Discussion

The laboratory and semi-field tests indicate that the MS-300 monitor is a reliable and efficient trap for *Ae*. *albopictus* and *Cx*. *quinquefasciatus*. Although it is a limitation that both species were not tested against each other, and the efficiencies of the MS-300 monitor in catching *Ae*. *albopictus* and *Cx*. *quinquefasciatus* in laboratory tests (98.5% and 95.8%) were higher than that in semi-field test (54.2% and 51.3%), no significant differences were found in the accuracy in the laboratory test (99.3% and 98.6%) and semi-field test (96.3% and 95.1%), which indicates that the captured mosquitoes in MS-300 are consistent with the data documented in cloud-based server. In the semi-field test, more than half of the mosquitoes were captured in the MS-300 with a lure but less than 5% in the MS-300 without a lure, which indicates that the MS-300 monitor with the Mix-5 lure can efficiently attract *Ae*. *albopictus* and *Cx*. *quinquefasciatus*.

With the real-time data from the semi-field tests, we can see that *Ae*. *albopictus* displayed distinct bimodal activity with a morning (8:00–10:00) and dusk peak (17:00–19:00), while only one activity peak (20:00–24:00) was observed for *Cx*. *quinquefasciatus*. These findings clarify the circadian behavior of *Ae*. *albopictus* and *Cx*. *quinquefasciatus* that might be used for guiding the mosquito control.

The field tests showed that the captured mosquito species are mainly *Ae*. *albopictus* and *Cx*. *quinquefasciatus*, which is consistent with previous reports [25, 26]. The numbers of *Ae*. *albopictus* and *Cx*. *quinquefasciatus* captured by the BGS traps were significantly higher than those of the MS-300 monitor, although both used the same Mix-5 lure. Possible reasons for the difference may be due to the appearance of BGS trap, which is more visually attractive to mosquitoes than the MS-300, and the air flowing in BGS traps may be more efficient in capturing mosquitoes than the MS-300 [17, 27], and these factors would be improved for a next-generation MS-300. However, there is no significant difference between the MS-300 monitor and BGS trap in the mean specificity in two field sites. The high accuracy capability of the MS-300 monitor shows that this internet-based device can accurately identify and record *Ae*. *albopictus* and *Cx*. *quinquefasciatus*.

Previous research has evaluated the effectiveness of the BG-Counter, a new internet-based device from BioGents [20, 28–32]. However, the effectiveness of BG-Counter varied greatly. Day *et al* assessed the BG-Counter in five North Carolina counties and the mean daily accuracy ranged from 9.4% to 80.1% [20]. One explanation for such low accuracy in the BG-Counter may be its one-way infrared LEDS and light detectors, which might not accurately discriminate objects passing through the detection window. The X- and Y-dimension infrared lights-detectors and lighting frequency design in MS-300 may increase the ability to discriminate the passing object and to identify the mosquitoes accurately. It may be verified in the further research of comparing the effectiveness and accuracy of the BG-Counter and the MS-300 monitor.

Temperature is the most important abiotic factor affecting development and survivorship of mosquitoes [33]. In the field tests, the number of *Ae*. *albopictus* captured by the MS-300 monitor showed a significant positive correlation with temperature at the two study sites. This supports the conclusion that the MS-300 monitor can be used to survey seasonal fluctuations of *Ae*. *albopictus* abundance indexed with the prevailing temperatures. The number of *Cx*. *quinquefasciatus* captured by the MS-300 monitor in the garden setting showed a significant negative correlation with temperature while the number was not significant in the residence. Seasonal variations in temperature and population density of these two vector mosquitoes recorded in real-time by the MS-300 monitor can serve as guide for mosquito control strategies under the differing conditions.

The success of MS-300 monitor was compared with human landing catches, the gold-standard method for monitoring mosquito density. The numbers of *Ae*. *albopictus* captured by the MS-300 monitor and BGS traps were correlated significantly with HLCs in both garden and residence trials. Meanwhile, the numbers of *Cx*. *quinquefasciatus* captured by the MS-300 monitor was correlated positively to those recovered in the BGS traps. HLCs were not considered in evaluating *Cx*. *quinquefasciatus* because HLCs were conducted from 17:30–18:30, which is not the period of peak activity for *Cx*. *quinquefasciatus*. Although the numbers of *Ae*. *albopictus* and *Cx*. *quinquefasciatus* captured by the BGS traps were higher than those of the MS-300 monitor, the high accuracy capability of the MS-300 monitor and positive correlation amongst these three monitoring methods means that the MS-300 monitor can inform users interested in monitoring circadian activity trends or determining the effectiveness of adult mosquito control efforts in an early warning manner.

## Conclusions

Our results demonstrate that the MS-300 monitor can be a dependable tool for assessing *Ae*. *albopictus* and *Cx*. *quinquefasciatus* population densities and circadian activity patterns in both laboratory and field surveys. It can be a powerful tool to guide prevention and control efforts of mosquito-borne diseases when applied to daily and seasonal population fluctuations and a potential surveillance tool for other mosquito species in other locations.

## Supporting information

S1 Fig Measurement of lengths of adult male and female *Aedes albopictus* and *Culex quinquefasciatus*. (PDF)

S1 Table Average lengths of adult male and female *Aedes albopictus* and *Culex quinquefasciatus*. (XLSX)

S2 Table Test of MS-300 monitor in laboratory. *Efficiency = number of mosquitoes in bag/number of mosquitoes released. †Accuracy = number of mosquitoes in bag/cloud-based server data.
(XLS)

S3 Table Test of MS-300 monitor in semi-field trail. *Efficiency = number of mosquitoes in bag/number of mosquitoes released. †Accuracy = number of mosquitoes in bag/cloud-based server data. ‡Correction formula of accuracy = 1-∣[1-(Number of mosquitoes in bag/cloud-based server data)]∣*100.
(XLS)
