# Supplementary figures and images for: Development and evaluation of an efficient and real-time monitoring system for the vector mosquitoes, Aedes albopictus and Culex quinquefasciatus
Source: PLoS Negl Trop Dis. 2022 Sep 8;16(9):e0010701. doi: 10.1371/journal.pntd.0010701 (PMC9455839; doi:10.1371/journal.pntd.0010701)

**A. *Aedes albopictus* ♂**

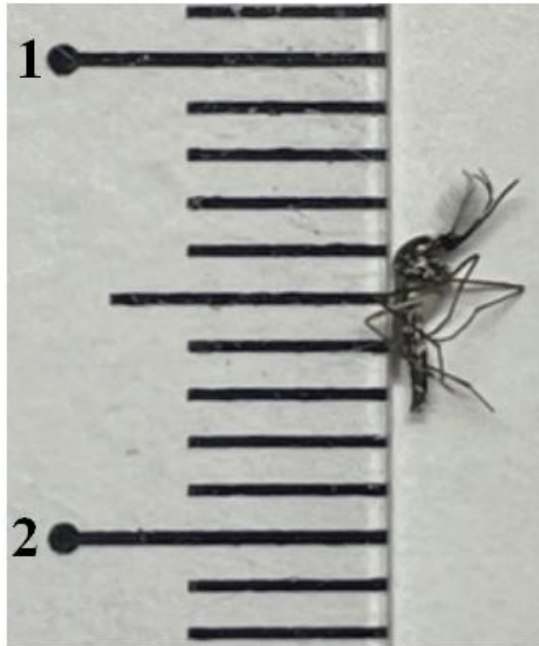

**B. *Aedes albopictus* ♀**

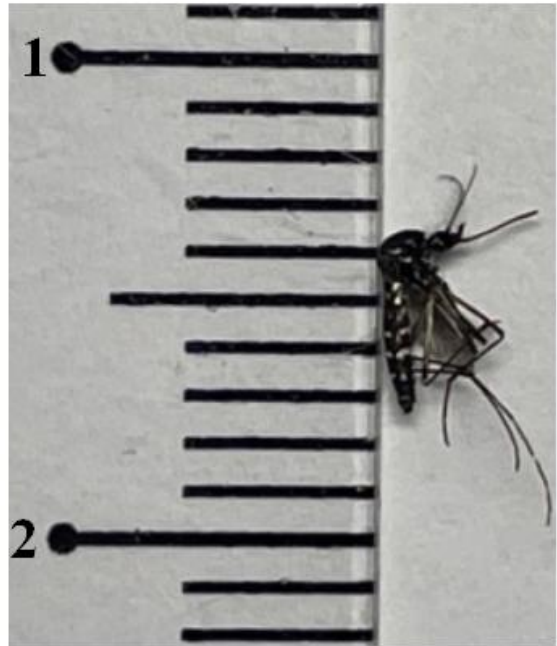

**C. *Culex quinquefasciatus* ♂**

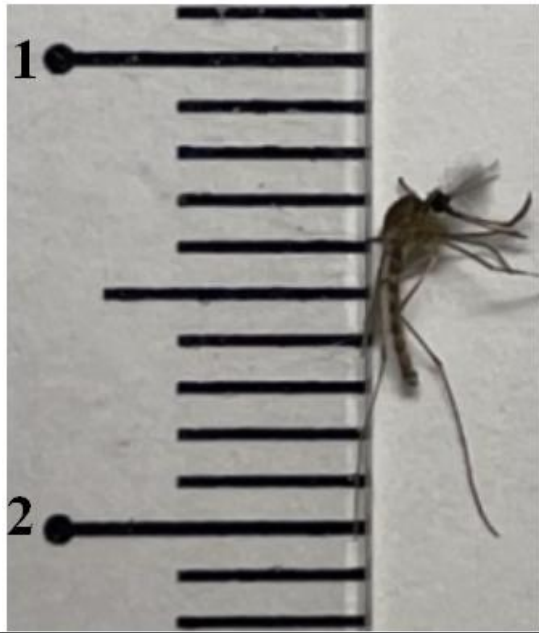

**D. *Culex quinquefasciatus* ♀**

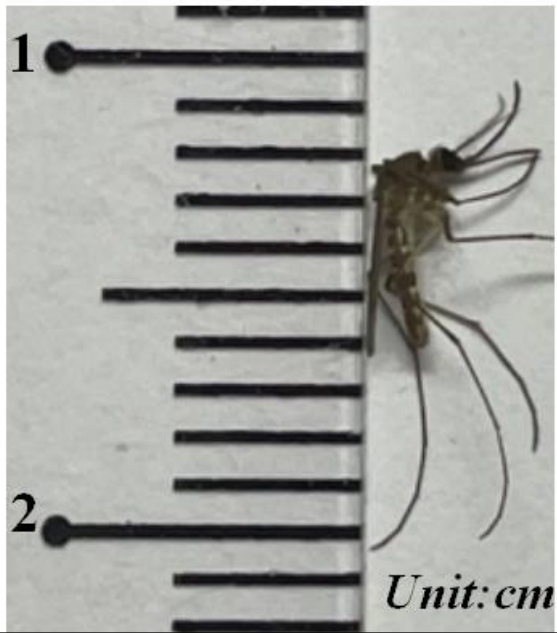

Supplement: S1 Fig — (PDF) [file pntd.0010701.s001.pdf]
